# Thermal Conductivity Enhancement of Polymeric Composites Using Hexagonal Boron Nitride: Design Strategies and Challenges

**DOI:** 10.3390/nano14040331

**Published:** 2024-02-07

**Authors:** Yuhang Meng, Dehong Yang, Xiangfen Jiang, Yoshio Bando, Xuebin Wang

**Affiliations:** 1 National Laboratory of Solid State Microstructures (NLSSM), Collaborative Innovation Center of Advanced Microstructures, Jiangsu Key Laboratory of Artificial Functional Materials, College of Engineering and Applied Sciences, Nanjing University, Nanjing 210093, China; 2 State Key Laboratory of Mechanics and Control of Mechanical Structures, Key Laboratory for Intelligent Nano Materials and Devices of the Ministry of Education, College of Material Science and Engineering, Nanjing University of Aeronautics and Astronautics, Nanjing 210016, China; 3 Chemistry Department, College of Science, King Saud University, Riyadh 11451, Saudi Arabia; 4 Australian Institute for Innovative Materials, University of Wollongong, Wollongong, NSW 2500, Australia

**Keywords:** hexagonal boron nitride (h-BN), thermally conductive polymeric composites, thermal conductivity, functionalization, thermal conduction networks

## Abstract

With the integration and miniaturization of chips, there is an increasing demand for improved heat dissipation. However, the low thermal conductivity (TC) of polymers, which are commonly used in chip packaging, has seriously limited the development of chips. To address this limitation, researchers have recently shown considerable interest in incorporating high-TC fillers into polymers to fabricate thermally conductive composites. Hexagonal boron nitride (h-BN) has emerged as a promising filler candidate due to its high-TC and excellent electrical insulation. This review comprehensively outlines the design strategies for using h-BN as a high-TC filler and covers intrinsic TC and morphology effects, functionalization methods, and the construction of three-dimensional (3D) thermal conduction networks. Additionally, it introduces some experimental TC measurement techniques of composites and theoretical computational simulations for composite design. Finally, the review summarizes some effective strategies and possible challenges for the design of h-BN fillers. This review provides researchers in the field of thermally conductive polymeric composites with a comprehensive understanding of thermal conduction and constructive guidance on h-BN design.

## 1. Introduction

In 1965, Moore proposed the famous “Moore’s Law”, which predicted that the number of transistors in an integrated circuit would double roughly every two years [1]. This not only reflects the rapid development of semiconductor technology but also reveals that electronic technology will progress toward a higher degree of integration with the acceleration of human information processing. Aligned with Moore’s prediction, contemporary electronic devices are evolving toward miniaturization, integration, and convenience. The trend of smaller, more concentrated devices places greater demands on their heat dissipation capabilities. Studies have shown that the performance of electronic equipment will decrease by 10% for every 2 °C increase [2,3]. Electronic packaging, which is an important part of combining various components of the chip, not only protects and connects integrated circuits but also plays an important role in the thermal management of integrated circuits. Unlike the thermal management requirements for materials like thermal barrier materials, which are between phase-change material and electrodes in phase change memory [4]; thermal conductivity (TC) switching materials in organic electronic devices [5]; and radiative cooling materials [6], the thermal management requirements of electronic packaging materials are focused on regulating the heat dissipation of the device. According to the types of materials used, the packaging of integrated circuits can be divided into plastic packaging, ceramic packaging, and metal packaging. Due to its excellent processability and cost-effectiveness, plastic has become a predominant choice for integrated circuit packaging, with over 90% of integrated circuits being encapsulated in plastic [7]. Plastics are mainly composed of polymers, but the lengthy chain structure and complex chain connection of polymers lead to the scattering of a large number of phonons, which results in the considerable inhibition of heat transfer in polymers, leading to their intrinsic low TC. This is not conducive to the heat dissipation of integrated circuits and has gradually become the bottleneck of the development of integrated circuits [8,9]. Therefore, improving the TC of packaging materials is of great benefit to the long-term, safe, and controllable operation of integrated circuit equipment, as well as the long-term development of the integrated circuit industry.

At present, a common method for synthesizing high-TC plastic packaging involves using a polymer as the matrix and adding fillers with high intrinsic TC to construct composites [10,11,12]. Fillers such as silver [13], graphene [14,15], carbon nanotubes [16], alumina [17], and hexagonal boron nitride (h-BN) [18] are commonly used. Among them, h-BN has attracted wide attention due to its high TC, wide band gap, and excellent electrical insulation properties [19,20,21]. In addition to h-BN, cubic boron nitride [22], cubic boron arsenide [23], and fluorinated graphene [24] have also been found to possess extremely high TC and excellent electrical insulation properties. However, their harsh preparation conditions and expensive raw materials make them costly and limit their widespread adoption [25,26,27]. The crystalline structure of h-BN comprises six-membered rings alternating between nitrogen and boron atoms within layers, with interlayer interactions mediated by van der Waals forces. It is often referred to as “white graphite” due to its structural resemblance to graphite [28]. In h-BN, heat is mainly conducted by phonons [29], and its unique structure reduces the phonon scattering caused by the disordered lattice in the material, resulting in high TC. At room temperature, the highest TC of bulk h-BN is about 400 W/(m · K), and the theoretical TC of a single-layer h-BN exceeds 600 W/(m · K) [30]. Notably, scholars have reported a remarkable TC value of up to 751 W/(m · K) for high-quality monolayer h-BN [25]. Morphology also has a significant impact on h-BN. For instance, theoretically, the TC of h-BN nanoribbons can reach 2067 W/(m · K) [31]. Moreover, h-BN typically exhibits an indirect band gap structure, as experimentally established at 5.955 eV using two-photon spectroscopy by Cassabois et al. [32]. Differing layers and various stacking arrangements have led to variation in its band gap within the range of 5–6 eV [33,34], and such a high band gap often leads to exceptional electrical insulation properties, which are instrumental in averting problems like current leakage and short circuits. At the same time, the density of h-BN is 2.27 g/cm^3^ [28], and due to this low density, the composites constructed with h-BN do not place a large burden on the device.

**Figure 1:**
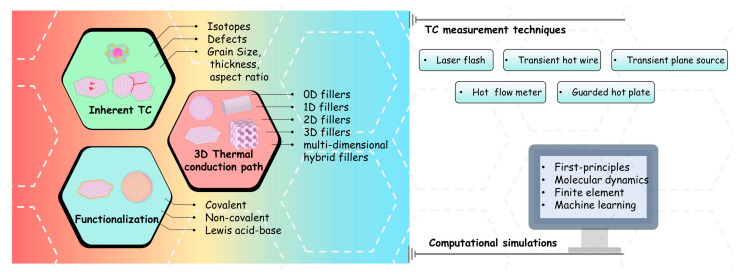
Schematic diagram of the design strategies of h-BN as a thermally conductive filler.

When using high-TC fillers to construct thermally conductive polymeric composites, it is necessary to consider the inherent TC and morphology of fillers, the interfacial resistance at the filler–polymer interface, and the thermal resistance among filler particles [35]. In line with these requirements, some of the design strategies for h-BN as a filler involve inherent TC and morphology, functionalization, and the design of three-dimensional (3D) thermal conduction networks. In this review, we present various design strategies for h-BN fillers that have emerged in recent years, along with common techniques for measuring the TC of composites. Concurrently, considering the advancement of information technology, computational simulation emerges as a pivotal tool, offering expeditious and precise guidance in the domain of h-BN designs. Therefore, some computational simulation methods are also briefly described. In conclusion, we present the effective strategies and potential challenges in designing h-BN for thermally conductive polymeric composites (Figure 1).

## 2. Heat Conduction Mechanism

### 2.1. Heat Conduction Model

Heat transfer can be divided into three modes: heat conduction, heat convection, and heat radiation. In solids, heat is mainly transferred in the form of heat conduction [36,37]. Fourier provided an empirical model for describing the heat change in this process [38], where the amount of heat passing through the unit area per unit of time is proportional to the temperature gradient in the direction normal to the plane, and the transfer occurs along the direction of decreasing temperature. It can be described with the following formula:

*J* = −*κ*∇*T* (1)

where *J* is the heat flux (W/m^2^), *κ* is TC (W/(m · K)), and *T* is the temperature.

Through a simple conversion of Equation (1), Equation (2) [36] can also be obtained:

*κ* = *αρC_p_* (2)

where *α* is the thermal diffusivity (m^2^/s), *ρ* is the density of the material, and *C_p_* is the specific heat capacity per unit volume of the material.

While Fourier’s law has been highly successful in describing macroscopic heat transfer, it does not always hold at the microscopic scale [39,40]. The inappropriateness of Fourier’s law mainly occurs when the characteristic scale of the material is comparable or even smaller than the mean free path of phonons, or when the time scale is comparable or smaller than the relaxation time of phonons [41,42]. Therefore, when calculating the TC of a composite, the formulas associated with Fourier’s law can still be used. For example, the laser flash method of measuring the TC of a material uses Equation (2). When analyzing TC at the nanoscale, other perspectives are necessary. To address the limitations of Fourier’s law, several new models have been proposed.

The Cattaneo–Vernotte (CV) model: To solve the infinite thermal conduction velocity problem of Fourier’s law, the CV model introduces a new parameter *τ_J_*, the lag time of the heat flow, which can be described with the following equation [43]:

(3) $$J+\tau_{J}\frac{\partial J}{\partial t}=-\kappa \nabla T$$

This model is a modification of Fourier’s law that takes into account the finite velocity of the heat wave. It is a very classical and typical model. Currently, this model is widely used in biomedicine, ultra-fast lasers, and electronic devices [42,43,44].

The dual-phase-lag (DPL) model: The DPL model was proposed by Tzou [45]. Compared to the CV model, the DPL model introduces another lag time for the temperature gradient, and two lag time parameters, *τ_J_* and *τ_T_*, are introduced. This leads to the possibility of not only solving the infinite thermal conduction velocity problem brought about by Fourier’s law but also describing the microstructural interactions such as phonon–electron interactions or phonon scattering [46,47]. Its application in composites is focused on the analysis of the thermodynamic response of materials under transient thermal shock [48,49]. This model can be described with the following equation [50]:

(4) $$J+\tau_{J}\frac{\partial J}{\partial t}=-\kappa (\nabla T+\tau_{T}\frac{\partial }{\partial t}(\nabla T))$$

The Guyer–Krumhansl (GK) model: The CV model does not apply to very low-temperature conditions where ballistic transport is in effect, while the GK model, another model that combines Fourier diffusion and heat waves, can address the size effect [51,52,53]. This model can be described with the following equation [54]:

(5) $$J+\tau_{J}\frac{\partial J}{\partial t}=-\kappa \nabla T+l^{2}\nabla^{2}J$$

The phonon Boltzmann transport equation (BTE): In contrast to the CV, DPL, and GK models, the Boltzmann transport equation (BTE) is specifically tailored to depict the dynamics of heat transfer mediated by phonons. It excels in characterizing situations where phonon transport transcends mere diffusion, thereby making it particularly suitable for describing heat conduction at the nanoscale. The BTE has been used to theoretically calculate the thermal conductivity of h-BN and the factors affecting heat transfer in h-BN with different nanostructures [29,30,55,56]. This model can be described with the following equation [57]:

(6) $$\frac{\partial f}{\partial t}+v\cdot \nabla_{r}f+F\cdot \frac{\partial f}{\partial q}=({\frac{\partial f}{\partial t})}_{coll}$$

where *f* is the distribution function, *t* is time, ***v*** is the phonon group velocity, ***r*** is the position of the phonon, ***q*** is the momentum of the phonon, ***F*** denotes the external force, and $\left(\frac{\partial f}{\partial t}\right)$*_coll_* is the scattering term.

### 2.2. Phonon Scattering

The carriers of heat conduction in solids are generally electrons and phonons. Electron mobility within polymers is restricted, making phonon heat conduction the predominant mechanism [58,59]. Phonons are quantized expressions of lattice vibrations, and an ordered lattice is conducive to heat conduction. N. Burger et al. described the heat conduction of crystalline materials and amorphous polymers by using the pellet model [36]. More degrees of freedom in amorphous polymer chains make heat easily dispersed to other chains, resulting in low TC (0.1–0.5 W/(m·K)) [60,61]. Table 1 summarizes the TC of the polymers covered in this review. The phonon’s TC can be related to the group velocity and the mean free path of phonons according to the Debye Equation (3):

(7) $$\kappa =\frac{C_{p}\upsilon l}{3}$$

where *υ* is the group velocity of the phonon, and *l* is the mean free path of the phonon. The mean free path of phonons is affected by the lattice structure, defects, and grain boundaries of the material, so the reduction in the TC of the materials is often related to the following factors [36]:

- Phonon–phonon scattering

The scattering that occurs between phonons (i.e., lattice vibrations) can occur with different frequencies or vibration modes and is called phonon–phonon scattering. In this case, the interactions of two or more phonons may lead to the exchange of energy and momentum between them.

- Phonon–defect scattering

When phonons propagate through the crystal, they encounter defects in the lattice, causing phonons to be scattered, thereby changing the direction and velocity of their propagation. This scattering has an important effect on the TC of crystals.

- Phonon–interface scattering

This type of phonon scattering is caused by interfaces or disconnections that occur during material processing. In the composites, it mainly occurs between the filler and the matrix.

**Table 1:** TC of the polymers covered in this review.

Matrix	TC (W/(m · K))	Reference
cellulose nanofiber (CNF)	1.24	[62]
cellulose nanocrystal (CNC)	0.5	[63]
epoxy	0.197	[64]
polyvinyl alcohol and chitosan (PVA and CS)	0.3	[65]
polyvinyl alcohol (PVA)	0.15	[66]
carboxylated styrene–butadiene rubber (XSBR)	0.092	[67]
epoxy-terminated dimethylsiloxane (ETDS)	0.2	[68]
poly(N-isopropyl-acrylamide) (PNIPAM)	0.57	[69]
polyimide (PI)	0.22	[70]
bisphenol E cyanate ester (BECy)	0.27	[71]
polypropylene (PP)	0.22	[72]
aramid nanofiber (ANF)	0.13	[73]
styrene–butadiene rubber (SBR)	0.19	[74]
poly(methyl methacrylate) (PMMA)	0.22	[75]
carboxylated acrylonitrile–butadiene rubber (XNBR)	0.16	[76]
polycarbonate (PC)	0.23	[77]
poly(vinylidene fluoride) (PVDF)	0.19	[78]
waterborne polyurethane (WPU)	0.18–0.24	[79]
polyethylene (PE)	0.2–0.5	[80]
polyetheretherketone (PEEK)	0.24	[81]

### 2.3. The Thermal Conduction Path Theory and Thermal Percolation Theory

Numerous scholars have studied the heat conduction mechanism in polymeric composites, with the prevailing theories considered to be the thermal conduction path theory and the thermal percolation theory [82,83,84,85]. The foundational concepts of both theories revolve around the establishment of thermal conduction networks, providing invaluable insights for the development of thermally conductive composites [86].

- The thermal conduction path theory

The thermal conduction path theory is widely used. According to this theory, the incorporation of fillers within the polymer establishes a new path for heat conduction, facilitating the rapid transmission of phonons from one end of the composite to the other by minimizing scattering along this path. For low filler concentrations, the fillers are relatively isolated, leading to slight enhancements in TC. With the increase in the proportion of fillers, the TC of the composites will rapidly increase due to the formation of a continuous thermal conduction path.

- The thermal percolation theory

The thermal percolation theory, in polymeric thermally conductive composites, is used to describe and predict the mechanism for enhancing TC. When fillers in polymers form a continuous network, the TC of the composites increases dramatically. This phenomenon is similar to the behavior of electrical or fluid percolation, so it is called “thermal percolation”. Even though some scholars have still clearly observed percolation thresholds in their experiments [63,72,87,88,89], some composites do not exhibit any abrupt changes in TC. Thus, this theory remains a subject of debate [83].

## 3. Intrinsic TC and Morphology of h-BN

Because heat conduction primarily depends on lattice vibrations, an ideal h-BN material tends to exhibit high TC. However, the TC of synthesized h-BN is affected by various characteristics such as isotopes, lattice defects, grain size, and thickness. At the same time, when it is used as a filler, h-BN interacts with the matrix. As a result, h-BN morphology also has a discernible influence on the TC of the composites.

### 3.1. Isotopes

**Figure 2:**
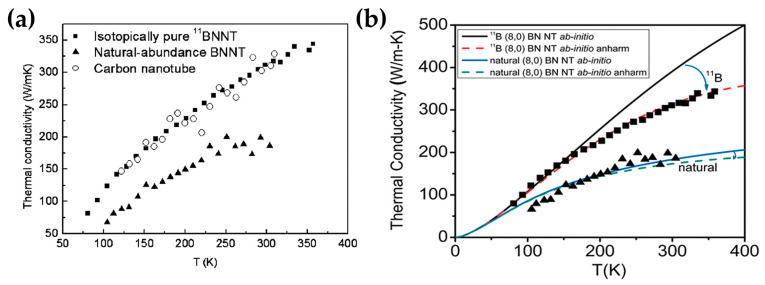
(**a**) The TC of a carbon nanotube (open circles), a BNNT (solid triangles), and an isotopically pure BNNT (solid squares) with similar outer diameters. Reprinted with permission from [90]. Copyright 2006, American Physical Society. (**b**) The TC for (8, 0) BNNT, 1 μm long, is shown as a function of temperature. The calculated TC with scaling factor for isotopically pure ^11^B and natural abundance BNNTs without anharmonic effects are shown as solid black and blue lines, respectively. The calculated TC with anharmonic scattering is denoted by dashed red and green lines for isotopically pure and natural abundance cases, respectively. Black squares and black triangles, respectively, denote experimental values for isotopically pure ^11^B and natural abundance h-BN nanotubes from ref [63]. Reprinted with permission from [92]. Copyright 2009, American Chemical Society.

Isotopes have a significant impact on some physical properties of materials. Although substances composed of different isotopes have the same electronic structure, alterations in the number of particles lead to variations in the lattice vibration mode. Chang et al. observed the TC changes caused by ^11^B substitution in h-BN nanotubes (BNNTs) using a microfabricated test fixture that could be directly characterized by transmission electron microscopy. The results showed that the TC of BNNTs with 99.5% ^11^B can be increased by 50% compared with natural abundance boron (i.e., 19.9% ^10^B and 80.1% ^11^B) (Figure 2a) [90]. Sevik et al. used the Einstein relation to conduct equilibrium molecular dynamics simulation and found that with the increase in the number of neutrons in the B atom, the TC of h-BN composed of ^8^B, ^9^B, ^10^B, and ^11^B increased in turn [91]. At the same time, the degree of the order and disorder of isotopes also has an impact on TC. Just like impurities, atoms of different isotopes will generate phonon isotopic scattering. Stewart et al. used the atomistic Green’s function method based on first principles to derive the force constant and processed isotope scattering according to a simplified cascade scattering model. In this way, they reproduced the experimental results of Chang et al., proving that isotope scattering strongly suppresses phonon transmission in high-frequency optical phonon bands (Figure 2b) [92]. Sevik et al. also studied the relationship between the TC of h-BN and the isotope concentration of ^10^B. The results showed that TC was the highest in the case of a single isotope and the lowest when the concentrations of ^10^B and ^11^B in h-BN were the same [91].

### 3.2. Defects

**Figure 3:**
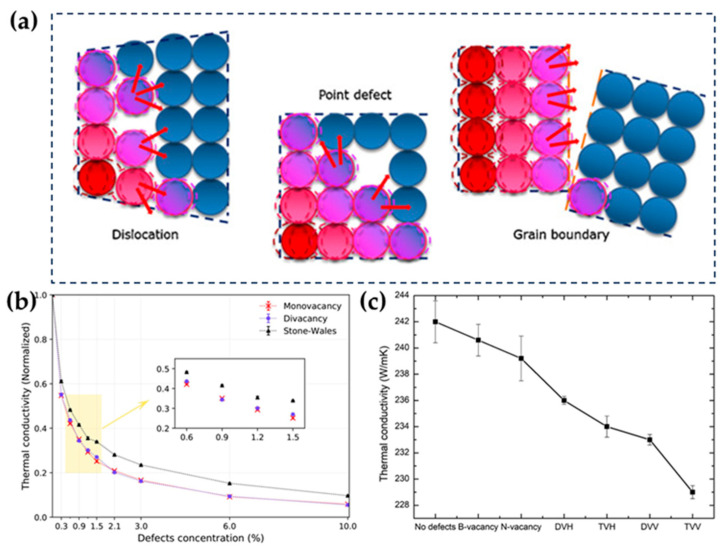
(**a**) Phonon scattering in crystalline materials, due to various defects (The color change from red to blue represents the lattice vibration from strong to weak, and the arrows represent the direction of heat transfer). Reprinted with permission from [36]. Copyright 2016, Elsevier. (**b**) The relationship between the normalized TC and the defect concentrations of three types of defective h-BN (monovacancy, divacancy, and Stone–Wales defects) with a size of 40 nm × 40 nm at 300 K. It includes nine defect concentrations of 0.3%, 0.6%, 0.9%, 1.2%, 1.5%, 2.1%, 3%, 6%, and 10%. Reprinted with permission from [93]. Copyright 2020, Elsevier. (**c**) TC of hydrogenated h-BN nanosheets with various vacancy defects (zigzag h-BN was used for no-vacancy defects). Reprinted with permission from [95]. Copyright 2023, Taylor & Francis.

Phonon scattering generated as a result of defects has a strong weakening effect on the TC of h-BN (Figure 3a). With the increase in defect concentration, the TC of h-BN will decrease significantly [93,94]. In addition to defect concentration, different defect types have different effects on the TC of h-BN. Wu et al. studied the effects of different defect types on single-layer h-BN, and the results showed that single- and double-vacancy defects led to a greater reduction in TC than Stone–Wales defects (Figure 3b) [93]. Additionally, considering the different electronegativity between the constituent elements (B and N), B and N vacancies have distinct impacts on the TC of h-BN. h-BN with an N-vacancy defect exhibits lower TC than that with a B-vacancy defect due to the fast-moving phonons around the N atom (Figure 3c) [95].

### 3.3. Grain Size, Thickness, and Aspect Ratio

According to the relationship between the phonon mean free range and the characteristic length of the crystal, phonon transport can be divided into ballistic and diffusive transport [96,97]. In addition, a large amount of phonon scattering occurs at grain boundaries [83]. This implies that the grain size has an obvious influence on the TC of composites. From the perspective of the fillers themselves, certain materials, such as silicon and diamond, exhibit an increase in TC with an increase in the grain size. When the grain size increases to a certain value, their TC approaches that of a single crystal. This is due to grain boundary and size effects [98]. However, the effect of grain size on the TC of h-BN is different. Chang et al. studied the change in the TC of BNNTs when the outer diameter changed from 30 to 40 nm using theoretical calculation, and their results showed that the TC decreased with the increase in the outer diameter [90]. Through computational simulations, Lu et al. also found that the TC of single-layer boron nitride nanosheets (BNNSs) decreases as the size increases. The reason is that as the simulated size increases, the inhibitory effect on TC caused by the increased scattering between phonons exceeds the contribution of the increased number of phonons to TC [99]. From the perspective of the composites, the small size of fillers results in the generation of more interfaces when forming the entire path, and the continuity of heat conduction decreases. The resulting Kapitza resistance is huge, which is not conducive to improving the TC of the composites [100,101]. During the formation of thermal conduction paths, a large-size filler has a smaller specific surface area and less interface in contact with the polymer, which leads to the formation of continuous heat conduction paths, effectively reducing phonon–interface scattering and improving the TC of the material [102,103,104]. Kim et al. studied the influence of different particle sizes on the TC of h-BN/ETDS composites, and the results showed that at the same loading, TC increased with the increase in particle size [68]. Moradi et al. also found that for fillers of different sizes (2, 30, and 180 μm), the TC of the composites increased as their size increased [105]. Therefore, the size of the fillers needs to be comprehensively considered in the synthesis of the composites.

**Figure 4:**
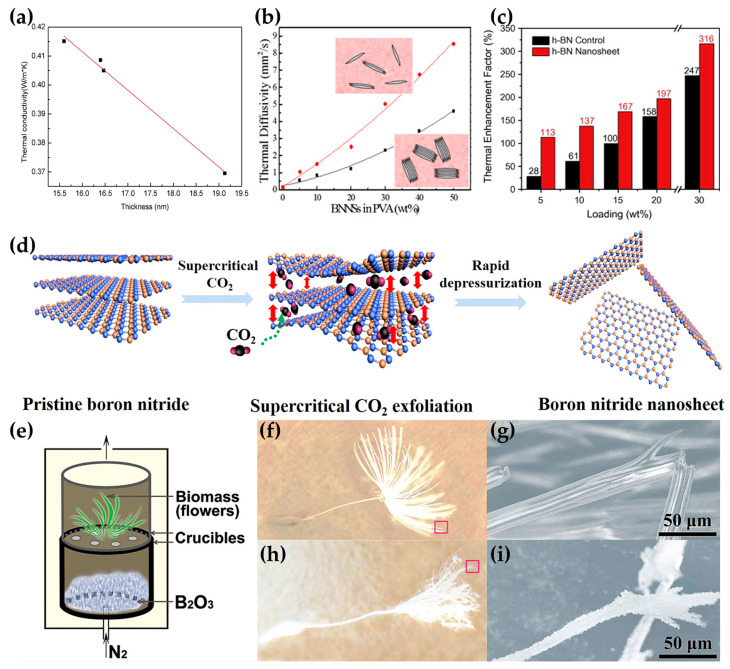
(**a**) Dependence of TC of BNNS/silicone rubber composites on BNNS thickness. Reprinted with permission from [108]. Copyright 2019, Elsevier. (**b**) In-plane thermal diffusivities of PVA nanocomposite films with microplasma-treated and untreated h-BN at different weight loadings [109]. This publication is licensed under CC-BY. Copyright 2016, American Chemical Society. (**c**) Thermal enhancement factors of BNNT-based composites and h-BN control-based nanocomposites. Reprinted with permission from [110]. Copyright 2013, Elsevier. (**d**) Scheme of BNNS synthesis using shear-assisted supercritical CO_2_ exfoliation [111]. This article is licensed under a Creative Commons Attribution 4.0 International License. Copyright 2017, Springer Nature. (**e**) Experimental scheme for biomass-directed on-site synthesis of BNNTs. Reprinted with permission from [19]. Copyright 2014, American Chemical Society. (**f**,**g**) A dandelion parachute and scanning electron microscopy (SEM) image of a pappus fiber with barbs. Reprinted with permission from [19]. Copyright 2014, American Chemical Society. (**h**,**i**) BN-nanosheet-assembled “dandelion parachute”. Reprinted with permission from [19]. Copyright 2014, American Chemical Society.

The thickness of h-BN is another factor affecting its TC. Its effect on the TC of the composites also consists of two main aspects. From the perspective of h-BN, based on the numerical solution of the phonon Boltzmann transport equation, Lindsay et al. theoretically estimated that the TC of monolayer h-BN at room temperature is greater than 600 W/(m · K), indicating that phonon–phonon scattering decreases with the reduction in the number of layers [30]. In 2019, Cai et al. calculated the TC of high-quality and clean monolayers of h-BN to be up to 751 W/(m · K) using the Raman technique [25]. For multilayer materials, both the intrinsic phonon–phonon scattering and out-of-plane vibration play an important role in determining the TC value. The interlayer interaction and coupling of multilayer h-BN break the two-dimensional selection rule, resulting in a significant decrease in TC compared with single-layer h-BN [30,55,106]. From the perspective of the composites, a too-thick h-BN is not conducive to the formation of thermal conduction paths [107]. Thinner h-BN has a lower percolation threshold. In the process of compounding BNNSs with silicone rubber, Lin et al. found that TC decreased linearly with the increase in the thickness of the nanosheets in the range of 15.5–19.5 nm (Figure 4a) [108]. Zhang et al. found that composites constructed from either single or several layers of h-BN obtained through microfluidic treatment exhibited a TC twice as high as that of untreated h-BN with more than 30 layers (Figure 4b) [109]. Lin et al. showed that the thermal enhancement factor of BNNSs was 113% at a loading of 5 wt%, while that of the bulk h-BN was only 28% (Figure 4c) [110]. Tian et al. prepared BNNSs with an average thickness of six layers using exfoliation assisted by supercritical CO_2_ shear forces. The TC of BNNS/epoxy composites improved by 313% (Figure 4d) [111]. Wang et al. prepared highly crystalline and atomically thin BNNSs by using the method of biomass-directed on-site synthesis, and the TC of BNNS/epoxy composites was 14 times higher than that of pure epoxy (Figure 4e–i) [19].

In essence, a filler with a large aspect ratio (defined as the ratio of transverse size to thickness) has a larger contact area with other fillers, leading to a lower interface density and less phonon scattering. Yan et al. obtained BNNSs with a high aspect ratio of 1500 by using microfluidization technology to exfoliate h-BN. They systematically studied the TC of composites with a length-to-diameter ratio and found that, at the same loading, fillers with a large length-to-diameter ratio resulted in more impressive TC improvement in the composites [112]. At the same time, materials with large aspect ratios can form a broader long-range order under the action of shear flow, which facilitates the construction of an effective thermal conduction path [113,114].

## 4. Functionalization of h-BN

The most important property of polymeric thermally conductive composites is their overall TC. The uniform dispersion of h-BN fillers is beneficial to the formation of internal thermal conduction networks in these composites, which eventually improves their TC. However, achieving optimal contact and uniform dispersion of pristine h-BN fillers in polymers presents challenges. This is primarily due to the smooth and chemically inert surface of h-BN, as well as the π–π interactions between h-BN layers, resulting in weak interactions between the h-BN fillers and the polymer, as well as the aggregation of BNNSs during the composite’s formation. This leads to the inevitable formation of gaps or voids between h-BN and the polymer. Since the TC of air within these voids is quite low, considerable phonon scattering occurs at the two-phase interface, which hinders the formation of efficient thermal conduction networks. Furthermore, the aggregation of h-BN leads to severe stress concentration [115,116], adversely affecting the overall mechanical performance of the composites. The effective way to solve this problem is to functionalize h-BN, which can be divided into covalent functionalization, non-covalent functionalization, and Lewis acid–base interaction according to the interaction between h-BN and functional molecules.

### 4.1. Covalent Functionalization

Covalent functionalization is a functionalization method that involves the use of active groups to chemically adsorb h-BN. According to different functional groups, functionalization can be mainly divided into two categories. One category is functionalizing h-BN with functional molecules (such as hydroxyl (OH) and amino (NH_2_) groups), and the other is functionalizing it with coupling agents (such as silane coupling agents). Hydroxyl and amino groups can improve the wettability of h-BN and enhance the interface compatibility between the h-BN and the polymers, which in turn reduces phonon–interface scattering and improves the TC of the composites [117,118]. There are many active sites for functionalization at the edges of h-BN due to the existence of unsaturated chemical bonds and the non-equilibrium state of atoms. In comparison, in-plane functionalization will break the complete lattice structure of h-BN and generate redundant phonon scattering, so edge functionalization is often a better choice for the functionalization modification of h-BN [119,120]. For example, Wu et al. prepared BNNSs using in-plane hydroxyl functionalization (POH-BNNSs) and edge functionalization (EOH-BNNSs) and then constructed BNNS/CNF composites. The results showed that the TC of POH-BNNS/CNF only exhibited a small increase (10.2%) at low BNNS loading and even a decreasing trend at high loading, while the TC of EOH-BNNS/CNF exhibited a 97.8% increase at a loading of 60 wt% h-BN [121].

Functionalization during the exfoliation of h-BN using ball milling is a common means for the surface hydroxyl functionalization of h-BN. The huge shear force during the ball milling process can exfoliate h-BN, reduce its thickness, and increase its inherent TC. At the same time, the huge energy during the ball milling process assists in the surface functionalization of h-BN [122,123]. However, the huge energy during the ball milling process inevitably destroys and disturbs the crystal structure, which reduces the TC of the h-BN itself. An effective approach to alleviating this issue is to perform ball milling in a solvent medium. The presence of a solvent medium serves to counteract the significant impact forces of mechanical ball milling and reduces shear forces, thereby preserving the integrity of the h-BN crystal structure to the greatest extent possible. Lee et al. used aqueous NaOH as a ball-milling auxiliary agent to promote the formation of hydroxyl on h-BN. The average size of BNNSs prepared using this method could reach 1.5 μm, and the hydroxyl content was as high as 18% [124]. Some scholars have also explored a similar approach with reduced power to moderate the huge energy of ball milling. For example, Chen et al. used high-temperature pretreatment and mild glucose-assisted mechanical agitation to functionalize BNNSs. High-temperature pretreatment reduces the interlayer force and preserves the lattice structure of BNNSs, while the excellent natural lubricity of glucose buffers against the strong shear force during the agitation process and further makes the hydroxyl group attached to the active site of BNNSs. At a BNNS loading of 20 wt%, the TC of BNNS/PVA and CS composites could reach 38.21 W/(m · K) [65].

**Figure 5:**
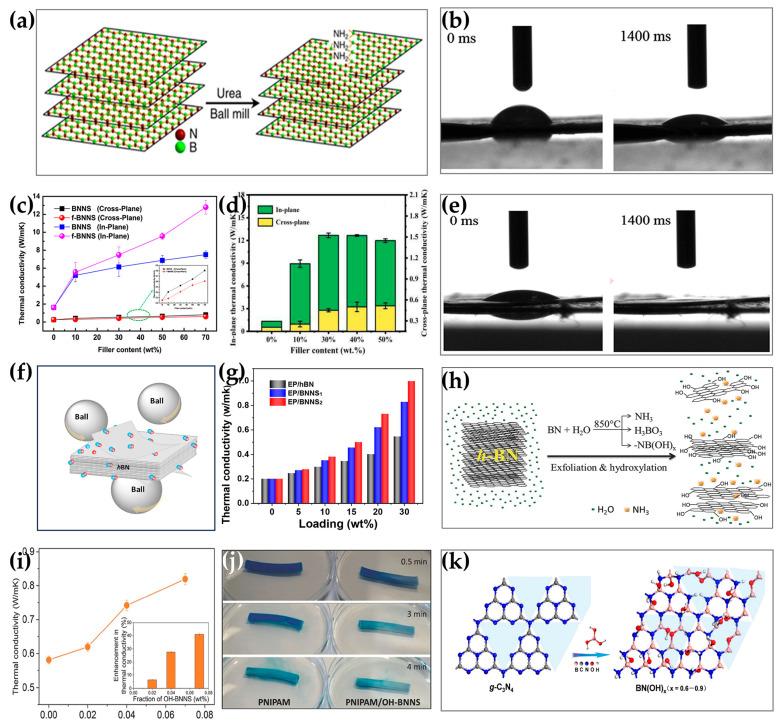
(**a**) Schematic illustration of amino-group functionalization via urea-assisted ball milling [123]. This work is licensed under a Creative Commons Attribution 4.0 International License. Copyright 2015, Springer Nature. (**b**,**e**) Results of water contact angle for (**b**) BNNSs and (**e**) f-BNNSs. Reprinted with permission from [119]. Copyright 2017, American Chemical Society. (**c**) In-plane and cross-plane TC of CNF composite films. Reprinted with permission from [119]. Copyright 2017, American Chemical Society. (**d**) In-plane and cross-plane TC of CNF composite films. Reprinted with permission from [115]. Copyright 2022, Elsevier. (**f**) Schematic illustration for exfoliation and functionalization of BNNSs using ball milling. Reprinted with permission from [125]. Copyright 2020, Elsevier. (**g**) TC of h-BN/epoxy composites. Reprinted with permission from [125]. Copyright 2020, Elsevier. (**h**) Schematic illustration of hydrolysis-assisted exfoliation and hydroxylation of h-BN powder in hot steam, where H_2_O reacts with h-BN, forming NH_3_ and –NB(OH)_x_, and the highly energetic H_2_O and NH_3_ assist the exfoliation through diffusion between h-BN layers. Reprinted with permission from [69]. Copyright 2015, John Wiley and Sons. (**i**) TC improvement with the increasing ratio of OH-BNNS (inset: improvement by percentage). Reprinted with permission from [69]. Copyright 2015, John Wiley and Sons. (**j**) Dye release upon heating, which resulted from the excellent integration between the OH-BNNS and H2O [69]. (**k**) Schematic illustration of the thermal substitution reaction between g-C_3_N_4_ and boric acid. Reprinted with permission from [131]. Copyright 2014, American Chemical Society.

As far as amino-group functionalization is concerned, ball milling is also a commonly used method. Lei et al. obtained functionalized h-BN rich in an amino group via urea-assisted ball milling (Figure 5a) [123]. Wang et al. also used urea as a modification agent in the ball milling process to fabricate amino-functionalized BNNSs. After functionalization, the TC of BNNS/epoxy composites constructed with amino-functionalized BNNSs was 0.384 W/(m · K), which was 3.5% higher than that of non-functionalized BNNSs [64]. Wu et al. used a similar method to prepare amino-rich BNNSs, and the hydrophilicity of the functionalized BNNSs was significantly improved. When they were combined with CNF, the in-plane TC of BNNS/CNF reached 30.25 W/(m · K) at a BNNS loading of 70 wt% (Figure 5b,c,e) [119]. In the process of ball milling, it is often possible to functionalize hydroxyl and amino groups of h-BN at the same time by selecting suitable modification reagents. Xu et al. used xylose-assisted ball milling to functionalize the hydroxyl and amino groups of h-BN and constructed h-BN/CNF composites. At a h-BN loading of 30 wt%, the TC of composites reached 12.68 W/(m · K), which was 846% higher than that of pure CNF (Figure 5d) [115]. Han et al. used (NH_4_)_3_PO_4_ and NaOH as milling additives to introduce hydroxyl and amino functional groups into BNNSs. The functionalized BNNSs formed a strong hydrogen bonding effect with the epoxy matrix, improving the dispersion of the filler in the matrix. At a BNNS loading of 30 wt%, the TC of the composites consisting of modified fillers reached 0.999 W/(m · K), indicating an increase of 397% (Figure 5f,g) [125].

In addition to ball milling, plasma treatment, steam flow treatment, water bath ultrasound, and acid/alkali/hydrogen peroxide-assisted ultrasound [126,127,128] are also commonly used for the hydroxyl and amino-group functionalization of h-BN. Xu et al. found that N_2_ activation led to an improvement in the amino-group functionalization of h-BN. At a h-BN loading of 50 wt%, the in-plane TC of h-BN/PVA reached 4.8 W/(m · K), and the through-plane TC of h-BN/PVA reached 2.4 W/(m · K) [18]. Xiao et al. performed the exfoliation of BNNSs with hot steam and realized the functionalization of hydroxyl groups. The hydroxyl group of BNNSs formed a hydrogen bond with PNIPAM hydrogel, resulting in an excellent dispersion in the PNIPAM matrix. By adding only 0.07 wt% of BNNSs, the TC of BNNS/PNIPAM composites increased by 41% (Figure 5h–j) [69]. Fu et al. first prepared hydroxyl-functionalized BNNSs in NaOH and KOH solutions via hydrothermal treatment, followed by ultrasonication, and they found that hydroxyl was more easily combined with the B atom [120]. In addition to strong bases, some scholars also used strong acids and the strong oxidation of hydrogen peroxide to achieve the hydroxylation of h-BN [129,130]. Weng et al. proposed a more interesting method to prepare BNNSs with extremely high hydroxyl content, in which boric acid is directly substituted for graphitic carbon nitride (g-C_3_N_4_) at the atomic scale under high-temperature conditions (Figure 5k) [131].

**Figure 6:**
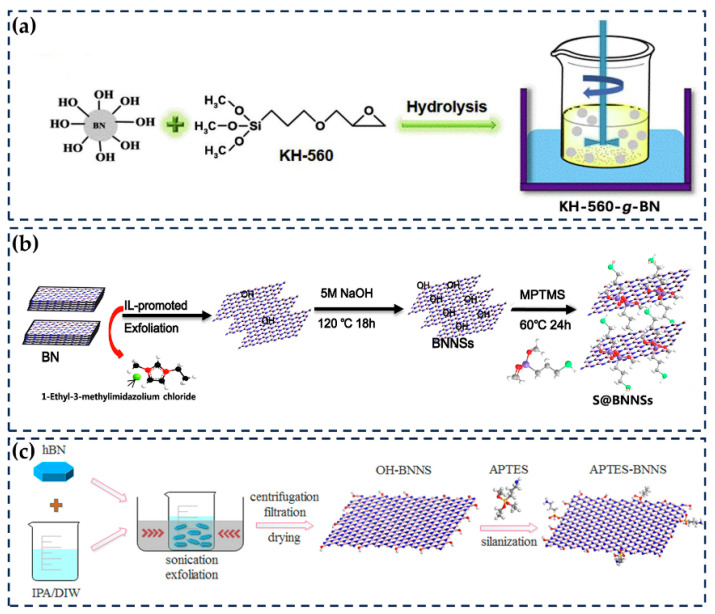
Schematic illustration of h-BN grafting macromolecular groups: (**a**) KH-560. Reprinted with permission from [132]. Copyright 2019, Elsevier. (**b**) MPTMS. Reprinted with permission from [67]. Copyright 2022, Elsevier. (**c**) APTES. Reprinted with permission from [107]. Copyright 2020, American Chemical Society.

Hydroxyl groups and amino groups in h-BN are relatively easy to functionalize. The method of grafting silane coupling agents onto these groups, although more complex, has also been widely studied [63,74]. The larger functional groups involved in this method hinder the stacking of fillers in the matrix and promote further dispersion and the formation of filling networks (Figure 6a) [129,132]. For example, Zhang et al. investigated S-based scion grafting to BNNSs via a two-step method: they introduced hydroxyl groups to the surface of BNNSs using NaOH and then vigorously stirred with 3-mercaptopropyltrimethoxysilane (MPTMS). When compounded with XSBR, at an MPTMS-BNNS loading of 47 wt%, the TC of the composites was 0.66 W/(m · K), 617.39% higher than that of pure XSBR (Figure 6b) [67]. Kim et al. used hydroxylated h-BN to graft 3-glycidoxypropyltrimethoxysilane (KBM-403) and 3-glycidoxypropyltrimethoxysilane (KBM-703) and then compounded them with ETDS. At a 70 wt% h-BN loading, the TC values of the composites were 4.11 and 3.88 W/(m · K), respectively. Compared with the TC of h-BN without surface treatment, with a value of 2.92 W/(m · K), the improvement is significant [68]. Gu et al. used h-BN to graft γ-aminopropyl triethoxy silane (KH550) and compounded it with epoxy. At a KH550-h-BN loading of 60 wt%, the TC value was 1.052 W/(m · K), five times higher than that of pure epoxy [133]. The grafted groups that can react with the matrix to form bridging groups on the surface of h-BN significantly contribute to the improvement in the TC of the composite [134]. Liu et al. grafted BNNSs with (3-aminopropyl)triethoxysilane (APTES). APTES was first adsorbed onto the surface of BNNSs via hydrogen bonds and then dehydrated with hydroxyl groups to form covalent bonds to achieve the surface functionalization of BNNSs. Then, the functionalized BNNSs were compounded with the epoxy. The TC of the APTES-BNNS/epoxy composites was up to 5.86 W/(m · K) at an APTES-BNNS loading of 40 wt%, which was 93.4% higher than that of h-BN/epoxy. The hydroxyl groups in APTES reacted with epoxy to connect BNNSs and epoxy and thus reduce the interface thermal resistance (Figure 6c) [107].

Compared to fillers without functionalization, covalently functionalized h-BN fillers can increase the TC of composites by 5–90%, which is a very considerable enhancement (Table 2). In addition, besides enhancing the TC of the composites, they can also improve the mechanical properties of the composites [135]. Li et al. constructed films using hydroxyl-functionalized BNNSs with PVA/CS. At a BNNS loading of 20%, the tensile strength of the composite doubled to 128.3 MPa compared to the pure PVA/CS film [38]. The enhancement in tensile strength is attributed to the fact that functionalization strengthens the interaction between BNNSs and the matrix, which results in a uniform arrangement of BNNSs and facilitates a payload transfer between the interfaces [65]. However, the tensile strength tends to decrease due to local stress concentration in the composites at high h-BN loading [107,119]. Additionally, since boron nitride has high compressive strength, fabricating composites with polymers can also enhance the compressive strength of polymers [136].

**Table 2:** Comparison of TC improvement via covalent functionalization.

Matrix	Modification Methods	Loading	Unmodified TC (W/(m · K))	Modified TC (W/(m · K))	Enhancement (%)
CNF [121]	Hydroxylation	60 wt%	12.27	24.27	97.8
epoxy [64]	Amino-group functionalization	10 wt%	0.28	0.38	3.5
epoxy [125]	Hydroxylation and amino-group functionalization	30 wt%	0.55	1.00	81.8
PVA [18]	Amino-group functionalization	50 wt%	1.50	2.40	60.0
XSBR [67]	MPTMS	47 wt%	0.57	0.66	15.8
ETDS [68]	KBM-403	70 wt%	2.92	4.11	40.8
ETDS [68]	KBM-703	70 wt%	2.92	3.88	32.9
epoxy [133]	KH550	60 wt%	0.99	1.05	5.8
epoxy [107]	APTES	40 wt%	3.03	5.86	93.4
CNF [119]	Amino-group functionalization	70 wt%	7.52	12.79	70.1
PI [137]	KR-44	50 wt%	0.74	0.86	16.2
epoxy [138]	HBP	5 wt%	0.27	0.33	22.2
epoxy [129]	APTES	30 wt%	1.04	1.18	13.5
PI [132]	KH-560 and NH_2_-POSS	30 wt%	0.69	0.71	2.9
PVA [139]	Hydroxylation	50 wt%	9.06	14.6	61.1

### 4.2. Non-Covalent Functionalization

Non-covalent functionalization is generally a functionalization method that uses molecules to physically adsorb on the h-BN surface. Compared with the huge energy required for covalent functionalization, non-covalent functionalization requires less energy, thus reducing the risk of introducing defects in the h-BN itself and maintaining its inherent TC as much as possible [76,140]. Physical adsorption in non-covalent functionalization occurs as a result of van der Waals forces, hydrogen bonds, π–π interactions, hydrophobic interactions, and electrostatic interactions.

**Figure 7:**
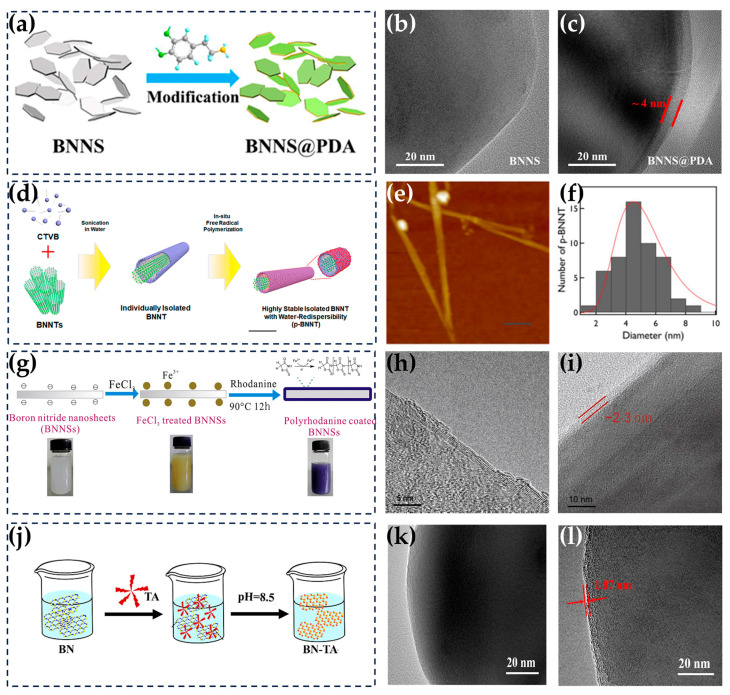
(**a**) Schematic illustration of PDA non-covalent functionalization. Reprinted with permission from [73]. Copyright 2020, American Chemical Society. (**b**) TEM images of BNNSs and (**c**) BNNSs and PDA. Reprinted with permission from [64]. Copyright 2020, American Chemical Society. (**d**) Schematic illustration of CTVB non-covalent functionalization. Reprinted with permission from [143]. Copyright 2020, American Chemical Society. (**e**) AFM images (0.5 μm × 0.5 μm) and (**f**) diameter distribution of functionalized BNNTs. Reprinted with permission from [143]. Copyright 2020, American Chemical Society. (**g**) Schematic illustration of PRh non-covalent functionalization. Reprinted with permission from [74]. Copyright 2016, Elsevier. (**h**) TEM images of BNNSs and (**i**) BNNSs and PRh. Reprinted with permission from [65]. Copyright 2016, Elsevier. (**j**) Schematic illustration of TA non-covalent functionalization. Reprinted with permission from [76]. Copyright 2021, American Chemical Society. (**k**) HR-TEM images of h-BN and (**l**) h-BN and TA. Reprinted with permission from [67]. Copyright 2021, American Chemical Society.

Different from covalent functionalization, non-covalent functionalization oftentimes occurs on the entire surface of h-BN instead of just the edges [141]. A common approach involves leveraging the spontaneous polymerization process of organic molecules to coat the h-BN surface with other organic molecules that exhibit excellent affinity, enhancing their affinity with the polymer matrix. Polydopamine (PDA) has good adhesion to both inorganic and organic molecules and is a commonly used non-covalent functional substance, with the method also known as the PDA coating method (Figure 7a–c) [71,72,73,142]. At a pH of 8.5, dopamine tends to spontaneously polymerize and coat the surface of h-BN through π–π interactions. PDA coating can significantly improve the dispersion of h-BN in the matrix, optimize the interface contact between the two phases, and facilitate the formation of thermal conduction paths. Nonetheless, the dopamine amorphous layer with low TC hinders heat conduction and may have poor compatibility with non-polar and hydrophobic matrices, thereby potentially reducing the overall TC when compounded with an untreated matrix, as shown in Table 3. In addition to dopamine, substances such as cetyltrimethylammonium 4-vinylbenzoate (CTVB), polyrhodanine (PRh), and tannic acid (TA) have been used in a similar scheme to achieve the non-covalent functionalization of h-BN. For instance, CTVB polymerizes in situ on the surface of BNNTs, driven by hydrophobic interactions, which ensures a high degree of stability for a long time and can easily disperse BNNTs in water even after freeze-drying (Figure 7d–f) [143]. Wu et al. used Fe^3+^ adsorbed on BNNSs as an initiator and oxidant to perform the in situ polymerization of PRh on the surface of BNNSs through hydrogen bonding and π–π interactions. The presence of PRh inhibited the joining of BNNS fillers in the matrix, resulting in good dispersion. At a BNNS loading of 27.5 vol%, the TC of BNNS/SBR composites increased by 36.3% compared with that without surface functionalization (Figure 7g–i) [74]. Similar to PDA, TA also oxidizes and self-polymerizes to oligomers at pH 7.8, coating BNNSs through physical interactions, which is faster and more cost-effective than PDA. Simultaneously, the abundance of phenolic hydroxyl groups on TA can promote its connection with the polymer matrix and reduce the interface thermal resistance (Figure 7j–l) [76].

In addition to polymer assembly coating, some scholars have also studied the methods involving the non-covalent functionalization of h-BN by using hydrogen bonds and charge interactions alone. Yu et al. calculated the differential charge density using density functional theory and revealed that the hydrogen bond between the nitrogen-containing polymer and h-BN can form relatively a strong interaction, which provides a new possibility for the non-covalent functionalization of the h-BN surface. They chose polyhexamethylene guanidine (PHMG) as a bridging agent to bridge h-BN and PVA through hydrogen bonding. As a result, the TC of the composites fabricated with functionalized h-BN as a filler was increased by eight times compared with the composites constructed with non-functionalized h-BN [141]. Wang et al. designed non-covalently functionalized h-BN with cationic polymer polyacrylamide (CPAM) by taking advantage of the negative charge caused by the electronegativity differences between B and N when h-BN is dissolved into water [77]. In addition, some ionic liquids can interact with h-BN through their charge characteristics and facilitate the non-covalent functionalization of h-BN, among which anion/cation–π interaction plays an important role in improving the functionalization ratio [144,145]. Morishita et al. used 1-butyl-3-methylimidazolium hexafluorophosphate ([bmim][PF_6_]) for the non-covalent functionalization of BNNSs and then compounded them with PMMA. With a BNNS loading of 24.5 wt%, the through-plane TC of the composites increased by 86.5% [145].

**Table 3:** Comparison of TC improvement via non-covalent functionalization.

Matrix	Modification Methods	Loading	Unmodified TC (W/(m · K))	Modified TC (W/(m · K))	Enhancement (%)
BECy [71]	PDA	15 vol%	0.55	0.50	−9.1
PP [72]	PDA	25 wt%	0.48	0.43	−10.4
ANF [73]	PDA	50 wt%	3.33	3.94	18.3
SBR [74]	PRh	27.5 vol%	0.55	0.75	36.3
PMMA [75]	CAS	80 wt%	3.81	10.22	168.2
XNBR [76]	TA	30 vol%	0.36	0.42	16.7
PC [77]	CPAM	20 wt%	0.62	0.73	17.7
PMMA [145]	[bmim][PF_6_]	24.5 wt%	0.52	0.97	86.5

### 4.3. Lewis Acid–Base Interaction

In addition to covalent and non-covalent functionalization, another method for the functionalization of h-BN involves a Lewis acid–base interaction [146,147]. The N atoms in h-BN are electron-rich, while the B atoms are electron-deficient, allowing h-BN to act as either a Lewis base or acid, interacting with certain substances through a Lewis acid–base reaction. Although this interaction is also non-covalent, it possesses strongly ionic characteristics [75].

At present, among the studies on the Lewis acid–base functionalization of h-BN, there are many studies on using B atoms as Lewis acid for functionalization. The amino groups in octadecylamine (ODA) or amine-terminated oligomeric polyethylene glycol can donate electrons and act as a Lewis base to interact with h-BN [148]. The typical process for the functionalization of h-BN with ODA involves mixing h-BN and ODA in a round-bottomed flask at a certain ratio (1:10 or 1:20), treating them with nitrogen at 160–180 °C for 96–144 h, cooling the mixture, adding tetrahydrofuran, and finally filtering or centrifugation [138,149]. At the same time, h-BN with more defect sites is more prone to ODA functionalization [150]. Furthermore, some scholars have simultaneously bifunctionalized h-BN to improve its compatibility with the matrix. Lee et al. used pyrene-tethered poly(4-vinylpyridine) (P4VP-Py) to bifunctionalize BNNSs with Lewis acid–base and π–π interactions. Additionally, bifunctionalized BNNSs exhibited higher dispersion in alcoholic solvents than tert-butyl and amino-functionalized BNNSs [151].

## 5. The Development of Thermal Conduction Networks of h-BN

**Figure 8:**
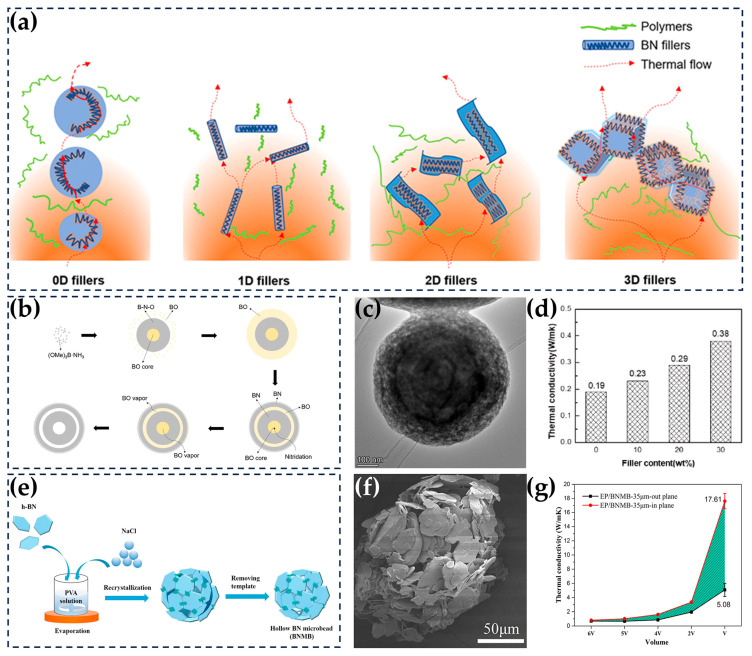
(**a**) Models of thermal transports in polymeric composites using fillers with different dimensions. Reprinted with permission from [153]. Copyright 2018, Elsevier. (**b**) Formation mechanism diagram of spherical precursor and onion-like cavitation structure of BNMS. Reprinted with permission from [156]. Copyright 2023, Elsevier. (**c**) TEM images of BNMS. Reprinted with permission from [156]. Copyright 2023, Elsevier. (**d**) TC of the BNMS/epoxy composites. Reprinted with permission from [156]. Copyright 2023, Elsevier. (**e**) Schematic diagram of the synthesis of hollow boron nitride microbeads (BNMBs). Reprinted with permission from [157]. Copyright 2019, Elsevier. (**f**) SEM images of BNMB, 35 μm. Reprinted with permission from [157]. Copyright 2019, Elsevier. (**g**) TC of BNMB/epoxy composites. Reprinted with permission from [157]. Copyright 2019, Elsevier.

According to the thermal conduction path theory of thermally conductive polymeric composites, heat is conducted along the 3D networks formed by the high-TC fillers in the composites. The effectiveness of the network has a decisive influence on the TC of the composites. Ensuring their processability, creating a more efficient network, and enhancing heat conduction with low h-BN loading are the goals of developing highly thermally conductive composites [89,152]. Herein, the fillers constituting 3D thermal conduction networks can be categorized based on their dimensions, namely zero-dimensional (0D), one-dimensional (1D), two-dimensional (2D), 3D, and multidimensional hybrid structures (Figure 8a).

### 5.1. Zero-Dimensional (0D) Fillers

As early as 1988, h-BN particles were used as fillers to improve the TC of polymers, which resulted in a relatively objective improvement of 11.7 times compared to the matrix [20,153]. Compared with multidimensional h-BN, 0D h-BN has unique advantages for reducing the viscosity of composites, achieving high levels of filling, and constructing isotropic composites [154,155]. Liu et al. prepared hollow h-BN microspheres (BNMSs) with a diameter of 0.8–3.4 μm through a chemical vapor deposition process involving trimethyl borate and ammonia. At a BNMS loading of 30 wt%, the TC of the BNMS/epoxy composite reached 0.38 W/(m·K) (Figure 8b–d) [156]. Xiao et al. constructed a hollow spherical h-BN skeleton using salt as a template and impregnated it using epoxy. At a h-BN loading of 65.6 vol%, the through-plane and in-plane TC values of the h-BN/epoxy composite reached 5.08 W/(m·K) and 17.61 W/(m·K), respectively (Figure 8e–g) [157]. Applying pressure during the curing process can improve the filler–matrix interface, increase the compactness of the composites, and improve the TC [158,159]. Moradi et al. studied the properties and behavior of composites under pressure in detail. The results showed that the TC of h-BN/epoxy composites, fabricated at a curing pressure of 58.3 MPa, increased from 3 W/(m · K) to 7 W/(m · K) as the density of the composites increased from 1.55 g/cm^3^ to 1.72 g/cm^3^ [160].

### 5.2. One-Dimensional (1D) Fillers

**Figure 9:**
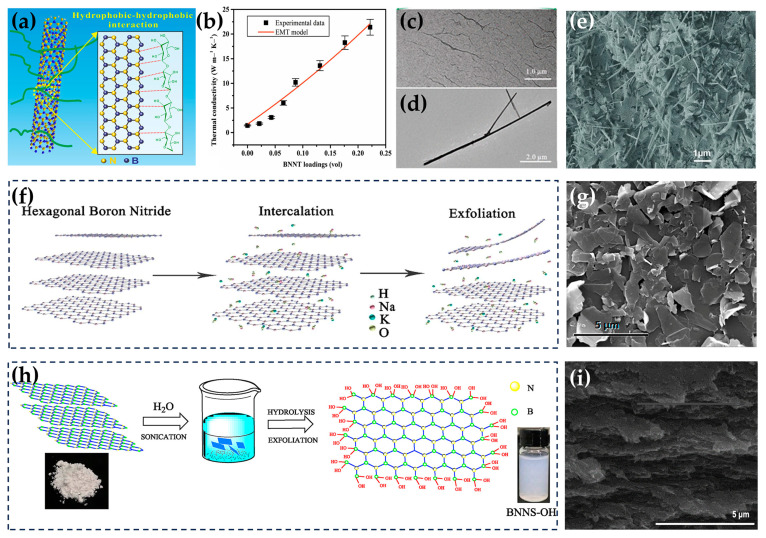
(**a**) Schematic of interaction between CNFs and BNNTs via hydrophobic–hydrophobic interaction. Reprinted with permission from [89]. Copyright 2017, American Chemical Society. (**b**) TC of BNNT/CNF nanocomposites. Reprinted with permission from [89]. Copyright 2017, American Chemical Society. (**c**,**d**) TEM images of (**c**) CNFs and (**d**) BNNTs. Reprinted with permission from [89]. Copyright 2017, American Chemical Society. (**e**) FE-SEM images of POSS-BNNT/epoxy nanocomposites. Reprinted with permission from [162]. Copyright 2013, John Wiley and Sons. (**f**) Schematic diagram of the exfoliation process. Reprinted with permission from [78]. Copyright 2018, Elsevier. (**g**) SEM image of BNNSs exfoliated from h-BN powder. Reprinted with permission from [78]. Copyright 2018, Elsevier. (**h**) Preparation of BNNS-OH. Reprinted with permission from [163]. Copyright 2018, Elsevier. (**i**) SEM image of cross-sectional BNNS-OH/CNF film. Reprinted with permission from [163]. Copyright 2018, Elsevier.

BNNT, a typical 1D filler in which heat conduction is completely dependent on phonons, is commonly used in applications with high TC that require electrical insulation [90]. Theoretically, the TC of a single-walled BNNT may exceed 3000 W/(m · K). Experimentally, the axial TC of BNNTs with a 40 nm diameter can reach 200–300 W/(m · K) [161]. Accurately utilizing the excellent TC of BNNTs in one dimension will be of great benefit in constructing highly thermally conductive composites at low filler loading [101,155]. Zeng et al. combined BNNTs with CNF. The BNNTs and their strong hydrophobic interaction with CNF laid the foundation for achieving good dispersion in CNF. At a BNNT loading of 25 wt%, the TC of BNNT/CNF composites reached 21.39 W/(m · K), nearly three times higher than that of the BNNS/CNF composites (6.9 W/(m · K)) at the same loading (Figure 9a–d) [89]. However, BNNTs have poor dispersion in high-viscosity thermosetting resins (such as epoxy), and this limits the improvement in the TC of the composites they build. To further improve the TC of BNNT/epoxy composites, Huang et al. performed surface functionalization by using polyhedral oligosilsesquioxane (POSS) grafted onto BNNTs (POSS-BNNTSs) with the assistance of hydroxyl and silane coupling agents. At a BNNT loading of 30 wt%, POSS-BNNT/epoxy exhibited a TC that was 13.6 times higher than that of pure epoxy (Figure 9e) [162].

### 5.3. Two-Dimensional (2D) Fillers

The key problem of constructing thermal conduction networks using two-dimensional BNNSs as fillers lies in the exfoliating, dispersion, and orientation of BNNSs. As described in Section 4.1, the ball milling exfoliation technique is a common method for fabricating BNNSs [163,164,165,166]. However, the huge shear force during ball milling also introduces defects into BNNSs, resulting in redundant phonon scattering. Moreover, the particle size of BNNSs obtained by ball milling is generally small, generating more interfaces during the construction of thermal conduction networks, which are not conducive to heat conduction [167]. In addition, biomass-directed techniques [19], molten hydroxide-assisted techniques [168], and ultrasonic exfoliation [164] are also widely used due to their high yield, mild organic solvents, and environmentally friendly benefits. Wang et al. used dandelion, fleabane flowers, and other biomass as carbon sources to perform the mass synthesis of high-quality and thin-layer BNNSs with an average thickness of 23 nm. Subsequently, they employed these nanosheets to fabricate composites with epoxy, resulting in a TC 14 times higher than that of pure epoxy [19]. Yu et al. fabricated BNNSs by mixing h-BN with hydroxide in a high-pressure reactor, heating the mixture, and then performing ultrasonic dispersion and filtration centrifugation. The yield of this method was as high as 19%. At a BNNS loading of 4 wt%, the in-plane TC of the BNNS/PVDF composites could reach 4.69 W/(m · K) (Figure 9f, g) [78]. Hu et al. directly exfoliated and prepared BNNSs using the ultrasonic method in an aqueous solution and then compounded them with CNF. The TC of the BNNS/CNF composite was as high as 22.67 W/(m · K) at a BNNS loading of 25 wt% [163] (Figure 9h,i).

**Figure 10:**
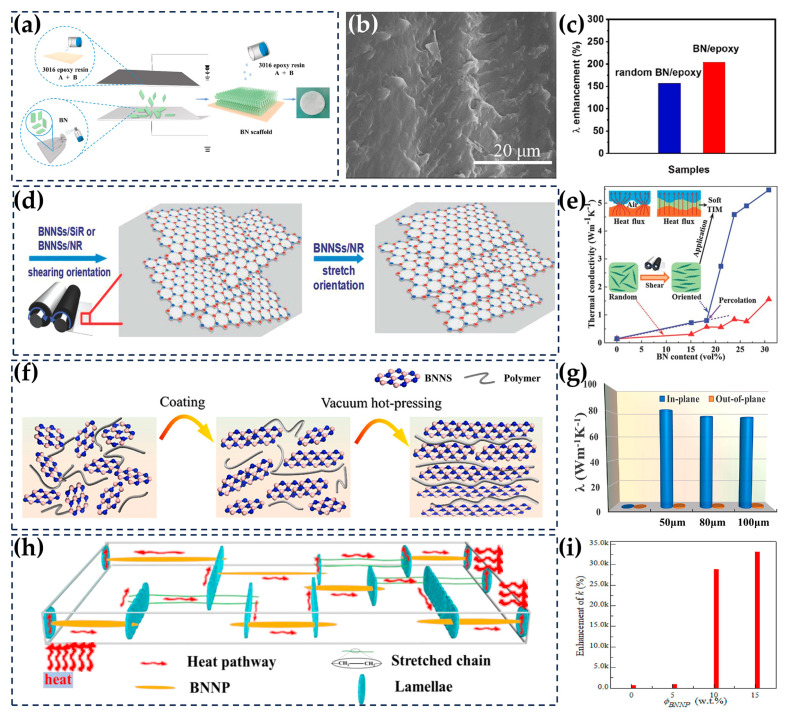
(**a**) Schematic representation of a procedure for preparing h-BN/epoxy composites via electrostatic flocking. Reprinted with permission from [170]. Copyright 2023, Elsevier. (**b**) Cross-section FE-SEM image of h-BN/epoxy composites via electrostatic flocking method. Reprinted with permission from [170]. Copyright 2023, Elsevier. (**c**) TC enhancement of composites in comparison to pure epoxy resins. Reprinted with permission from [162]. Copyright 2023, Elsevier. (**d**) Schematic diagram of BNNS orientation process. Reprinted with permission from [172]. Copyright 2014, John Wiley and Sons. (**e**) Comparison diagram of TC of composites composed of oriented BNNS fillers and random BNNS fillers. Reprinted with permission from [172]. Copyright 2014, John Wiley and Sons. (**f**) Schematic illustration of the formation process of the BNNSs with ultrahigh TC using directional coating formation and vacuum pressure technology. Reprinted with permission from [79]. Copyright 2022, Elsevier. (**g**) In-plane and out-of-plane TC of BNNSs with different thicknesses. Reprinted with permission from [79]. Copyright 2022, Elsevier. (**h**) Schematic showing potential thermal conduction mechanisms within the stretched nanocomposite film. Reprinted with permission from [80]. Copyright 2020, Elsevier. (**i**) Enhancement of TC for nanocomposite films. Reprinted with permission from [80]. Copyright 2020, Elsevier.

The effective dispersion of BNNSs is mainly achieved through surface functionalization, which was elaborated on in Section 4. In addition, the average TC of 2D BNNSs is higher than that of 0D nanoparticles and 1D nanotubes, since heat is more easily conducted along the in-plane direction. [19]. However, the crystal structure of h-BN is anisotropic, and its TC in the c-axis direction can be as low as 2 W/(m · K) [169]. Therefore, the orientation of BNNSs in the composites also has a profound effect on the overall thermal conduction properties of the material. On the one hand, adjusting the orientation can reduce the degree of anisotropy in the TC of the composites. Li et al. used the electrostatic flocking method to realize the vertical orientation of BNNSs. At a 17.57 wt% BNNS loading, the through-plane TC of BNNS/epoxy composites was 18.6% higher than that of a random orientation (Figure 10a–c) [170]. In addition to electric field forces, magnetic field forces have also been used to enhance the through-plane TC of composites [171]. On the other hand, adjusting the orientation can enhance the in-plane TC of the composites. Kuang et al. adjusted the orientation of BNNSs by introducing shear forces during the composite process. Compared with the random distribution of BNNSs, the oriented BNNSs improved the TC of the composites by 3.5 times (Figure 10d,e) [172]. In addition to shear forces, pressure and tensile forces have also been used by scholars to enhance the orientation of BNNSs in the matrix. Yu et al. realized the directional arrangement of BNNSs in the matrix through a two-step process involving directional coating formation and vacuum pressure technology. The resulting BNNSs were then compounded with epoxy and waterborne polyurethane (WPU). The in-plane TC of the prepared BNNS/epoxy and WPU composites was surprisingly high, at 81.49 W/(m · K) (Figure 10f,g) [79]. Zhang et al. prepared BNNS/PE composites with BNNSs having layered arrangement using uniaxial stretching technique, and their TC was as high as 106 W/(m · K) at a BNNS loading of 15 wt% (Figure 10h,i) [80].

### 5.4. Three-Dimensional (3D) Fillers

It is a desirable goal to achieve high TC under low filler loading and guarantee the processability of composites. Excellent 3D fillers can minimize the interfacial contact of fillers and have higher heat conduction efficiency per unit mass than randomly dispersed filler systems. [173,174,175]. They are expected to maximize the TC of composites with minimal loading [176], which is also an important direction for the design of highly thermally conductive composites.

**Figure 11:**
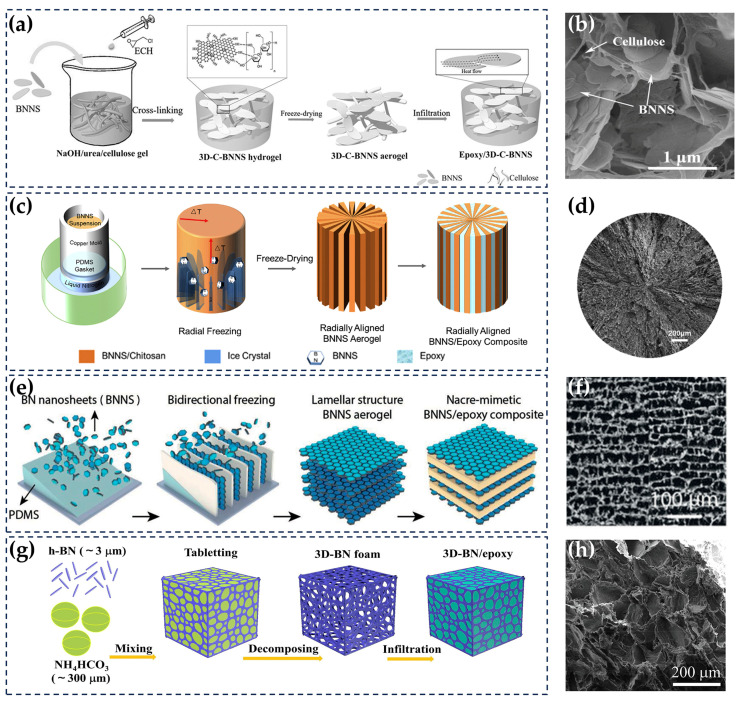
(**a**) Scheme illustrating the preparation process of 3D-C-BNNS/epoxy nanocomposites. Reprinted with permission from [175]. Copyright 2016, John Wiley and Sons. (**b**) SEM images of 3D-C-BNNS aerogel. Reprinted with permission from [175]. Copyright 2016, John Wiley and Sons. (**c**) The schematic diagram for the fabrication route of radially aligned BNNS/epoxy composites using a radial freeze-casting method. Reprinted with permission from [178]. Copyright 2020, Elsevier. (**d**) SEM images of BNNS aerogel. Reprinted with permission from [178]. Copyright 2020, Elsevier. (**e**) Schematic illustration of the fabrication route using a bidirectional freezing technique together with epoxy resin infiltration. Reprinted with permission from [179]. Copyright 2019, John Wiley and Sons. (**f**) The cross-sectional SEM images of BNNS aerogel. Reprinted with permission from [179]. Copyright 2019, John Wiley and Sons. (**g**) Sketch map depicting the formation process of the 3D-BN/epoxy composites. Reprinted with permission from [181]. Copyright 2020, Elsevier. (**h**) Cross-section SEM images of 3D-BN foams. Reprinted with permission from [181]. Copyright 2020, Elsevier.

Nagaoka et al. developed Cellulose and h-BN core–shell spherical microbeads via phase separation. Anisotropic composites were obtained by mixing core–shell spherical beads, epoxy resin, and curing agents and then using a centrifugal stirrer under a certain pressure. The h-BN interconnects on the surface of the microbeads acted as thermal paths, and the TC of the prepared composites with a 48.5 vol% h-BN loading reached 10.6 and 15.6 W/(m · K) for the through-plane and in-plane directions, respectively [177]. Similarly, Huang et al. also used cellulose and BNNSs to construct the thermal conduction network. The difference was that they used cellulose nanofibers as the skeleton to support BNNSs and finally prepared BNNS/epoxy composites with a 3D thermal conduction network via vacuum-assisted impregnation. The TC of BNNS/epoxy composites reached 3.13 W/(m · K) at a low loading of 9.6 vol%, which was approximately 14 times higher than that of pure epoxy (Figure 11a,b) [175]. In addition, designers often create a 3D filler skeleton and then impregnate it with epoxy to prepare epoxy-based composites. Chen et al. constructed a hashed BNNS thermal conduction network based on a temperature gradient using the radial freeze-casting method. After radially freeze-casting BNNSs and chitosan, the resulting structure was immersed in epoxy and heated to facilitate solidification, leading to the formation of the BNNS/epoxy composites. It had a through-plane TC of 4.02 W/(m · K) and an in-plane TC of 3.87 W/(m · K) at a low BNNS loading of 15 vol% (Figure 11c,d) [178]. Bai et al. adopted the bidirectional freezing technique to develop a layered distributed 3D BNNS network under a certain temperature gradient. This 3D BN network was immersed in epoxy resin to obtain a composite that had a high in-plane TC of 6.07 W/(m · K) at a BNNS loading of 15 vol% (Figure 11e,f) [179]. The template method is also a common method to construct a 3D h-BN [180]. Wang et al. built a 3D h-BN network by sacrificing NH_4_HCO_3_. It was impregnated with epoxy to prepare the composite. At a loading of 59.43 vol%, the interfacial TC of the composite reached 6.11 W/(m · K), about 34 times higher than that of pure epoxy (Figure 11g,h) [181]. Feng et al. used a melamine–formaldehyde sponge (MS) as a template to fabricate a composite. At a h-BN loading of 35.9 wt%, the through-plane and in-plane TC of the composite reached 4.95 W/(m · K) and 10.20 W/(m · K), respectively [182].

### 5.5. Multidimensional Hybrid Fillers

Multidimensional filler hybridization is also an effective strategy for building 3D networks [183,184,185,186]. Since 3D fillers have been used to build a fairly complete thermal conduction network, multidimensional filler hybridization often occurs between 0D, 1D, and 2D fillers. Compared with single-dimensional fillers, multidimensional filler hybridization has the characteristics of improving the dispersion of fillers, making it easier to bridge adjacent fillers to form a thermal conduction network and helping to reduce voids in composite materials, which results in a synergistic heat conduction enhancement [83]. Using hybrid fillers comprising 0D and 2D fillers is one of the common approaches. In one scenario, 2D fillers can fill the gaps between 0D fillers. Hong et al. found that when the ratio of aluminum nitride (AlN) and h-BN was 1:1, h-BN could fill the gaps between AlN, and the TC of the composites constructed using them was the highest [187]. In another scenario, 0D fillers served as bridges between 2D fillers [188,189]. Wang et al. found that due to the bridging connections of silver nanoparticles when the BNNS loading was 25.1 vol%, the TC of the composite increased from 1.63 to 3.06 W/(m · K) [190]. Using hybrid fillers composed of 1D and 2D fillers is another common approach [191,192], where 1D BNNTs can be used to promote bridging between 2D BNNSs. Su et al. found that compared with BNNT/epoxy and BNNS/epoxy composites, the TC values of BNNTs and BNNS/epoxy composites were higher at the same loading [193].

## 6. TC measurement Techniques and Computational Simulations

Measurement techniques and computational simulations are indispensable in modern engineering and scientific research, serving the dual purposes of understanding ‘what’ through various TC measurement techniques and unraveling ‘why’ through computational simulation analysis. TC measurement techniques allow for a precise understanding of how a material behaves, while computational simulation analysis provides insights into the underlying reasons for the observed behavior and even enables the prediction of future behavior. This section provides a brief overview of TC measurement techniques and computational simulation analysis in the context of thermally conductive composites.

### 6.1. TC Measurement Techniques

Currently, TC measurement techniques can be categorized into steady-state methods and transient methods.

#### 6.1.1. Steady-State Methods

The steady-state method is used to measure the process in which the temperature at each point on the specimen reaches equilibrium and does not change over time. This method is accurate and simple, but the measurement time tends to be longer. Commonly used steady-state methods include the heat flow meter method and the guarded hot plate method.

- The heat flow meter method

To measure the TC of a material using the heat flow meter method, a specimen is placed between a heat source and a cold source, and the temperature gradient and heat flux at both ends of the material are measured with temperature and heat flux sensors. This method can be used to measure the TC of glass, ceramics, polymers, and insulation materials [101,194]. The TC of the material can then be calculated using the following equation [195]:

(8) $$\kappa =\frac{JL}{\Delta T}$$

where *L* is the thickness of the specimen, and Δ*T* is the temperature difference between the upper and lower surfaces of the specimen.

- The guarded hot plate method

In the guarded hot plate method, a hot plate is sandwiched between two identical specimens. Additionally, a cold plate is placed above and below the entire component formed by the two specimens. The specimen is further protected by a surrounding protective heater, ensuring that the heat generated by the hot plate is effectively absorbed by the specimen. This method allows for the measurement of a narrower range of TC of materials and can be used to measure the TC of glass, polymers, and insulation materials [101]. The TC of the material can then be calculated using the following equation [196]:

(9) $$\kappa =\frac{QL}{2A\Delta T}$$

where *Q* is the power supplied, and *A* is the cross-sectional area of the plates.

#### 6.1.2. Transient Methods

The temperature of the specimen varies with time during the measurement using the transient method. This method is advantageous due to having less environmental burden and fast measurement speed. Commonly used transient methods include the laser flash method, the transient plane source method, and the transient hot wire method.

- The laser flash method

The laser flash method is used to measure the TC of a material by emitting an instantaneous pulse from a laser source, which uniformly irradiates the surface of the specimen. This causes the temperature of the surface layer to rise instantaneously as the energy is absorbed. This surface acts as the hot end and transfers the energy to the cold end (the opposite surface) through one-dimensional heat conduction. An infrared detector is used to continuously measure the temperature rise at the center of the cold end of the specimen, providing information about the material’s temperature response over time. The laser flash method is the most commonly used method to measure the TC of thermally conductive polymeric composites [63,65,74,75,78,110,134,138]. It is used to measure the thermal diffusivity of the material, which is obtained using Equation (9). The TC of the material can then be obtained using Equation (2).

(10) $$\alpha =0.1388\frac{L^{2}}{t50}$$

where *t*_50_ represents the time required for the temperature of the upper surface of the specimen to reach half of its maximum value.

- The transient hot wire method

In measurements using the transient hot wire method, a hot wire, functioning as both a heat source and a sensor, is inserted into a medium. The TC of the medium is determined by measuring the temperature rise in the hot wire as a function of time. The TC of the material can then be calculated using the following equation [197]:

(11) $$\kappa =\frac{qdlnt}{4\pi d\Delta T(t)}$$

where *q* is the thermal power per unit length.

- The transient plane source method

The transient plane source method, also known as the hot disk method [198,199], has some similarities to the hot wire method. During the measurement, a probe is positioned between two specimens, serving both as a heat source and a temperature sensor. The rate at which heat diffuses through a material is influenced by its thermal properties, including thermal diffusivity and TC. These properties can be determined by recording the temperature and response time of the probe. This method is fast, versatile, and capable of measuring TC up to an upper limit of 1800 W/(m · K). However, its accuracy diminishes when applied to materials with low TC [101,200,201].

### 6.2. Computational Simulations

With the rapid development of computing technology, the focus of material research has also shifted from experiments to computational simulations. The application of computational simulations in designing experimental schemes and predicting experimental results is booming. A burgeoning array of computational methods is being developed, accompanied by the development of increasingly sophisticated computational software. Currently, in the design and validation of thermally conductive materials, computational simulation also has a pivotal role, which provides scholars with the possibility of further improving and refining the thermal conduction performance.

**Figure 12:**
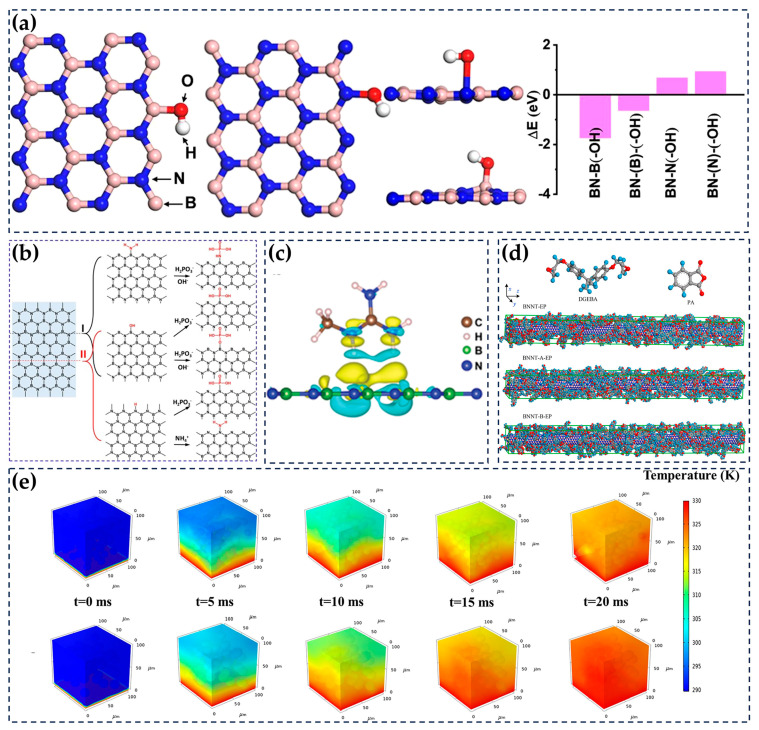
(**a**) Models of hydroxyl groups at different positions on the edge of h-BN and the enthalpies of reaction for the fractured h-BN layer with hydroxyl groups. Reprinted with permission from [65]. Copyright 2022, Elsevier. (**b**) Proposed chemical reaction mechanisms for functionalization of BNNSs. Reprinted with permission from [125]. Copyright 2020, Elsevier. (**c**) Differential charge–density diagram of guanidine adsorbed by h-BN. The yellow and blue regions indicate where the charge density increases and decreases, respectively [141]. This is an open access article distributed under the terms of the Creative Commons CC BY license. Copyright 2023, John Wiley and Sons. (**d**) Composite models of BNNT, DGEBA, and PA. Reprinted with permission from [203]. Copyright 2021, Elsevier. (**e**) Finite element simulation results of small-size h-BN (top) and large-size h-BN (bottom) composite material models. Reprinted with permission from [104]. Copyright 2022, Elsevier.

The concept of computational scale pertains to the utilization of various computational methodologies for simulating physical systems of varying sizes [202]. At the atomic and molecular levels, typically on the nanometer scale, first-principle calculation methods and molecular dynamics simulation methods are commonly employed. At macroscopic levels, typically in the millimeter range, finite element analysis methods are commonly adopted. These two scales and their corresponding calculation techniques demonstrate similarities in that they are all based on physical laws and mathematical models to describe the material properties and behaviors, but they are different in the values of the physical and computational quantities involved, as well as the phenomena and processes that can be simulated. At the atomic level, the first-principle density functional theory (DFT) is often used by scholars to analyze the surface functionalization of h-BN, which allows for the determination of adsorption sites and configurations of functional groups, such as OH and NH_2_, by calculating the minimal enthalpy change incurred during group adsorption onto h-BN (Figure 12a) [65,118]. For example, Han et al. used the DFT calculation method with the B3LYP function and determined that the enthalpy change in the bond between OH and NH_2_ functional groups and B at the edge of h-BN during the milling process is minimal. This implies that these functional groups readily bond with B, facilitating the functionalization of h-BN (Figure 12b) [125]. Yu et al. employed the DFT method to calculate the variation in the adsorption energy of methylguanidine on h-BN for different orientations and determined that methylguanidine can establish robust adsorption through N-H...N structure with h-BN through differential charge density (Figure 12c) [141]. Unlike DFT, molecular dynamics methods are more effective in simulating the interaction between functionalized fillers and the polymer matrix. Zhang et al. constructed two models of BNNTs: one in which C atoms replaced B atoms, followed by grafting NH_2_ groups (BNNT-A), and another in which the B-N bonds were broken, leading to B atoms being grafted with NH_2_ groups and N atoms forming bonds with H atoms (BNNT-B). Then, the two models were combined with diglycidyl ether of bisphenol A epoxy resin (DGEBA) and phthalic anhydride (PA) to build the composites, which were subsequently subjected to simulation. The results showed that the composite constructed with the BNNT-A model had a higher TC, up to 2.52 W/(m · K), which was increased by 77.5% compared with BNNT/epoxy, indicating that carbon doping is an effective scheme to improve the interface contact of fillers and enhance the TC of composites (Figure 12d) [203]. Oh et al. simulated the heat conduction behavior of the h-BN/PEEK composite using the molecular dynamics method. The filling of h-BN increased the phonon strength at 79 THz, and the elevated phonon vibration in the h-BN plate promoted heat conduction. At the same time, the addition of h-BN also promoted the wide distribution of polymer chains, which are conducive to the construction of long-range heat conduction paths [81]. At the macro level, finite element modeling can simulate information such as the filler shape, the matrix shape, and the filler distribution, and it has been widely used to predict the effective TC of polymeric thermally conductive composites [204]. Wang et al. established a 100 × 100 × 100 μm h-BN/HDPE composite model and used the finite element method to analyze the influence of h-BN fillers with different particle sizes on the overall TC of the composites. The results showed that large particle sizes facilitated the formation of thermal conduction paths and heat diffusion to the other end (Figure 12e) [104]. Xu et al. used a transient finite element simulation method to simulate the thermal conduction performance of composites. The results showed that compared with random fillers, the heat conduction of the composites constructed with 3D interconnected fillers was faster, proving that the construction of thermal conduction paths played a decisive role in the rapid conduction of heat [181].

Computational simulation is a numerical method based on physical models. In addition, data-driven machine learning methods are increasingly being applied to h-BN heat conduction [205,206]. For instance, Ding et al. achieved successful predictions of TC for composites at different loadings using a neural network with three hidden layers. The predictions were based on various parameters, including filler TC, matrix TC, temperature, and volume fraction. This underscores the potential of machine learning in advancing the comprehension of h-BN heat conduction [207].

## 7. Summary and Outlook

The unique in-plane structure of h-BN results in a very high in-plane TC, while its relatively wide band gap limits the excitation of electrons, thus exhibiting strong insulation properties. This presents promising prospects for the design of electronic packaging composites. However, the construction of high-TC composites is not an overnight achievement. The inherent TC of h-BN, dispersion within the matrix, and structural continuity within the matrix are the three factors that restrict the TC of the composites. Optimizing these aspects is a general approach for the design of high-TC composite materials. Several strategies can be considered simultaneously:

(1) To improve the inherent TC of h-BN, reducing phonon scattering is crucial. Selecting h-BN fillers with fewer defects and high crystallinity can help achieve this goal. Additionally, when selecting the morphology of h-BN, factors such as grain size, thickness, and aspect ratio of h-BN need to be considered comprehensively.
(2) To improve the dispersion of h-BN in a matrix, chemical or mechanochemical methods can be used to covalently functionalize its edges with hydroxyl, amino, or silane coupling agents to enhance its compatibility with the matrix. Alternatively, non-covalent functionalization through organic coating can be employed to achieve non-destructive surface modification without altering its internal structure.
(3) To construct continuous 3D thermal conduction networks, h-BN of various dimensions can be used. Compared to using 0D, 1D, and 2D h-BN as fillers, using 3D h-BN facilitates the formation of effective thermal conduction paths. Additionally, multidimensional hybrid fillers can also be used to control filler distribution and optimize paths.

With the rapid development of computational technologies, utilizing calculation and simulation techniques has become a crucial means of improving design efficiency, capability, and cost-effectiveness. In the construction of polymeric thermally conductive composites, simulations based on physical models play an important role in calculating the intrinsic TC of h-BN, the analysis of the functionalization mechanism of h-BN, and the structural design of h-BN in composites. In addition, data-driven machine learning is gradually being applied in the field of composite construction. With the support of a large volume of theoretical realization data, machine learning will provide new ideas for the rational design and preparation of high-TC composites.

Although some progress has been made in h-BN-filled polymeric thermally conductive composites, there are still some challenges in the current design. Firstly, the synthesis of large, high-quality, and ultrathin h-BN remains a difficult task. The tape mechanical exfoliation method is simple to operate but not suitable for large-scale applications. Liquid-phase exfoliation exhibits greater exfoliation efficiency, but the size, defects, etc., of BNNSs are difficult to control, and keeping impurities and defects at a low level is a challenge. Chemical vapor deposition is a method for preparing large-area, high-purity BNNSs but usually requires relatively high temperatures, which may adversely affect some substrate materials or device components and may also cause difficulty in controlling the crystal growth direction. Additionally, the high-precision design and synthesis of 3D networks in composites is still an issue. The template method is accurate, but suitable templates are difficult to prepare. Furthermore, limitations in processing performance and mechanical properties under high loading pose additional challenges in composite construction. In the future, with a combination of experiments and simulations, the solution to these problems should be explored to further develop the potential of h-BN-filled polymeric thermally conductive composites.
